# Expanding the investigation of meaningful effects in physiology research

**DOI:** 10.4155/fsoa-2017-0057

**Published:** 2017-07-07

**Authors:** Kevin Deighton, James A King, David J Stensel, Ben Jones

**Affiliations:** 1 Institute for Sport, Physical Activity & Leisure, Leeds Beckett University, Leeds, LS6 3QS, UK; 2 School of Sport, Exercise & Health Sciences, Loughborough University, Loughborough, LE11 3TU, UK

**Keywords:** appetite, confidence intervals, individual differences, magnitude-based inferences, nonresponders, personalized medicine, physiology, responders, significance testing, statistics

The statistical investigation of meaningful changes in response to physiological interventions has increased considerably during the past decade. Indeed, in the field of exercise physiology it is now commonplace for performance test outcomes to be assessed using magnitude-based inferences (MBI) as either the sole method of statistical analysis [1] or in combination with null-hypothesis significance testing [2]. Additionally, the focus on ‘personalized medicine’ during recent years has stimulated significant interest in the quantification of true and meaningful individual responses to interventions within the field of human physiology. The purpose of the present article is to provide a brief overview of MBI and individual response differences, with a focus on the potential for wider applications in other areas of physiology research. Recent developments from our research groups are used as examples to demonstrate the potential for an expanded use of these approaches.

## Investigating meaningful effects at the group level

The MBI method derives the probability that an effect is beneficial, harmful or trivial based on the observed effect and its uncertainty in relation to a predetermined value representing a minimum clinically or practically important value of the effect [3]. This differs from null-hypothesis significance testing which assesses the span of confidence intervals (CIs) in relation to a ‘null’ effect (i.e., if the CIs of the effect do not span zero then the effect is deemed ‘significant’). Rather than assessing significant differences, MBI provides an interpretation of the magnitude of changes and whether these are meaningful, which represents an intuitive approach for many researchers [4]. Assessing the magnitude of change in a probabilistic manner also reduces inferential error rates, increases the proportion of decisive (publishable) outcomes, and reduces publication bias, especially with small sample sizes [3].

The implementation of MBI for analysis of an intervention requires determination of a value for the smallest meaningful change in the relevant variable. To achieve this, it is often preferable to use a pre-established value informed by the literature which represents a practical or clinical benefit. Such values have been established for a range of variables in relation to minimum clinically important differences (e.g., the 6-min walk test in patients with chronic obstructive pulmonary disease [5]) or practical benefits (e.g., changes in athletic performance tests [6]). The recent incorporation of MBI to investigate changes in appetite perceptions in response to an acute exercise and nutritional intervention [7] utilized a well-established threshold for practically relevant changes of 8–10 mm when assessed using a 100-mm visual analogue scale [8]. This represents the first use of MBI in the analysis of appetite perceptions and highlights the potential wider utility of this approach in physiology research.

In addition to the approach described above, fractions of the between-subject SD may also be used as the value for the smallest meaningful change in the relevant variable (e.g., 20% of the between-subject SD would represent the threshold for a small effect size of 0.2 based on Cohen's *d*) [6]. This method represents a reasonable starting point for the assessment of novel variables in the absence of established meaningful change values of practical or clinical relevance.

## Investigating meaningful individual responses

In combination with the assessment of effects at the group level, investigations into individual response differences have become prevalent within physiology research. This approach typically classifies participants as either ‘responders’ or ‘nonresponders’ based on the direction or magnitude of their individual response to an intervention [9,10]. Further statistical analyses or additional research studies are then sometimes performed to elucidate the reasons for these divergent responses. For example, this may involve an investigation into the participant characteristics of ‘responders’ compared with ‘nonresponders’, or further investigations into the underlying physiology of these groups of participants. However, this approach to classifying individual response differences does not account for random within-subject variation, which is comprised of natural biological variation between measurement points and the technical error from the measurement tool/protocol [9,11,12]. In a recent publication, Atkinson & Batterham [9] provided a comprehensive overview of the potential influence of random within-subject variation on the measurement of physiological variables and demonstrated that this variation can sometimes account entirely for the apparent individual response differences observed. To remove the influence of random within-subject variation, true individual response differences require the SD of changes in response to an intervention to be greater than the same SD in a comparator arm (for randomized controlled trials) or from a prior reliability study (for crossover trials) [9]. The magnitude of this difference must be either practically or clinically relevant before mediators of this effect are to be examined [9].

The work of Atkinson & Batterham [9] has emphasized the need for researchers to understand the random within-subject variation for a range of physiological measures before attempting to investigate individual response differences. Considering that random within-subject biological variation is likely to increase as the time period between trials becomes longer [9,13], it is important that acute crossover studies utilize reliability data from investigations that have separated trials by a similar period of time. The recruitment of similar participant populations is also important to increase the relevance and accuracy of reliability data. Accordingly, reliability studies have recently been employed within appetite research to determine individual differences in the appetite and energy intake responses to exercise [14] and food consumption [15]. Additionally, the work by King *et al*. [14] determined the within-subject variation in plasma acylated ghrelin concentrations as a mechanistic variable for understanding changes in appetite perceptions. This focus to understand meaningful individual responses in mechanistic and primary outcome measures may represent a useful model for other areas of physiology research. These studies also highlight the topical nature of investigations to understand random within-subject variation to provide a platform for the accurate assessment of true and meaningful individual response differences. Further investigation of other physiological variables is required, in addition to the examination of whether individual responses remain stable with repeated exposures to an intervention [15,16].

## Conclusion & future perspective

MBI and the accurate quantification of individual response differences represent two recent statistical developments for the evaluation of physiological outcomes. The novel focus on these aspects of analysis in appetite research demonstrates the potential for more widespread use to assess a range of variables across a variety of research topics. Indeed, the integration of MBI within statistical analysis can be readily achieved by the determination of smallest meaningful change values as either a fraction of the between-subject SD or using established thresholds of practical or clinical relevance. Equally, with the increased focus on personalized medicine and nutrition, it is important for researchers to accurately assess true and meaningful individual response differences before conducting further research or providing a personalized intervention. We anticipate that the prevalence of these statistical approaches will increase in the coming years across a wider range of research topics.
